# Chromosome 1p13 genetic variants antagonize the risk of myocardial infarction associated with high ApoB serum levels

**DOI:** 10.1186/1471-2261-12-90

**Published:** 2012-10-16

**Authors:** Bruna Gigante, Karin Leander, Max Vikström, Shu Ye, Ulf de Faire

**Affiliations:** 1 Division of Cardiovascular Epidemiology, Institute of Environmental Medicine (IMM), Karolinska Institutet, Stockholm, Sweden; 2 Division of Cardiovascular Medicine, Department of Clinical Sciences, Danderyd Hospital, Karolinska Institutet, Stockholm, Sweden; 3 Department of Cardiology, Karolinska University Hospital, Stockholm, Sweden; 4 Centre of Clinical Pharmacology, William Harvey Research Institute, Barts and the London School of Medicine and Dentistry, Queen Mary University of London, London, UK

## Abstract

**Background:**

Genetic variation at 1p13 modulates serum lipid levels and the risk of coronary heart disease through the regulation of serum lipid levels. Here we investigate if the interaction between genetic variants at 1p13 and serum lipid levels affects the risk of non-fatal myocardial infarction (MI) in the Stockholm Heart Epidemiology Program (SHEEP), a large population based case control study.

**Methods:**

In the present study only non fatal MI cases (n = 1213, men/women: 852/361) and controls (n = 1516, men/women =1054/507) matched by age, sex and residential area, were included. Three SNPs 12740374 G/T, rs599839A/G and rs646776T/C mapping at 1p13 were analysed for association with serum lipid levels and the risk of MI by a weighted least square regression and logistic regression analyses, respectively. To analyse the effect of the interaction between genetic variants and serum lipid levels on the risk of MI, we applied the biological model of interaction that estimates the difference in risk, expressed as OR (95%CI), observed in the presence and in the absence of both exposures. One derived measure is the Synergy index (S) and 95%CI, where S > 1 indicates synergy and S < 1 antagonism between the two interaction terms.

**Results:**

Rs12740374G/T and rs646776T/C were in strong linkage disequilibrium (LD) (r^2^ = 0.99), therefore only rs599839A/G and rs646776 were included in the analysis. Consistently with published data, presence of the rare genotypes was associated with reduced total-, LDL-cholesterol and ApoB serum levels (all p < 0.05) as compared to the reference genotype, but was not associated with the risk of MI.

However, the increased risk of MI observed in individual exposed to high (≥75^th^ percentile) serum lipid levels was offset in subjects carrying the rare alleles G and C. In particular, the risk of MI associated with high ApoB serum levels OR (95%CI) 2.27 (1.86-2.77) was reduced to 1.76 (1.33-2.34) in the presence of the G allele at rs599839 with an S of 0.47 (0.20-0.90).

**Conclusions:**

These results indicate that an antagonism between ApoB serum levels and genetic variants at 1p13 contributes to reduce the risk of non-fatal MI in the presence of high ApoB serum levels.

## Background

Genome wide association studies (GWAS) performed in large international consortia have demonstrated that variation at chromosome 1p13 is associated with the risk of coronary artery disease (CAD) mainly through its association with LDL and cholesterol serum levels [1-6]. Two leading SNPs mapping at this locus rs646776T/C and rs599839A/G explain 1% of the genetic variation in circulating LDL-cholesterol levels and the rare alleles are associated with reduced LDL-cholesterol levels [5]. Chromosome 1p13 maps in close proximity to the cadherin EGF LAG seven-pass G-type receptor (*CELSR2*) and the proline/serine-rich coiled-coil protein 1 (*PSRC1*) genes, involved in the regulation of cell adhesion, proliferation and intracellular trafficking, and in proximity to the gene coding sortilin (*SORT1*) a cell surface receptor involved in the glucose and lipid uptake. Functional studies have shown that the genetic variants at this locus modulate cholesterol metabolism through the regulation of sortilin expression and LDL uptake in hepatocytes and influence the diameter of the circulating LDL particles [7,8].

The estimated risk [expressed as odds ratio (OR) and 95% confidence interval (95%CI)] of CAD in individuals carrying the allele associated with high LDL-cholesterol levels ranges from 1.20 (1.1-1.3) [6] to 1.29 (1.2-1.4) [9] and 1.19 (1.13-1.26) for the early onset myocardial infarction (MI) [10]. Consistently, the rs599839 G allele, associated with low LDL-cholesterol levels, was associated with a 13% 90%CI (10-17) reduction in the risk of CAD [7].

The actual effect of a genetic variant on the risk of complex diseases can vary across different studies [11] and populations depending on the genetic architecture, the outcome of the study and the exposure to different risk factors [12-14]. To overcome these limitations and fully explain the risk of cardiovascular diseases associated with these newly discovered genetic variants, different approaches have been proposed and applied. In particular, fine mapping of the region of interest [15], the analysis of the association with more specific traits and the analysis of gene and environment interactions [14] have been recently proposed to fill in the so called “missing heritability” gap.

Here we investigate if an interaction between variants at chromosome 1p13 and serum lipid levels was associated with the risk of non-fatal MI. We performed the present study in the Stockholm Heart Epidemiology Program, SHEEP, a large case control population recruited in the Stockholm area specifically designed to investigate the role of genetic and environmental factors in the occurrence of MI in men and women.

## Methods

### Study population

SHEEP [16] was designed as a population based case control study to dissect both genetic and environmental factors underlying the occurrence of MI and to compare the effects of the different risk factors in men and women. Cases were identified during the period 1992 to 1994. The sources were the coronary and intensive care units, the discharge charts from the hospitals in the Stockholm County area and the death certificates from the Swedish National Causes of Death Register. The criteria for myocardial infarction included changes in the CK and LDH blood levels, presence of specified ECG changes and/or the autopsy finding of a myocardial necrosis whose age was compatible with the time of disease onset. Only patients who survived at least 28 days after the MI event were included in the present study (n = 1213, men = 852; women = 361). One control per case was randomly selected from the Stockholm County population registry after stratification for age (with a 5-years interval), sex and residential area. In addition other 5 controls were selected at the same time to replace eventual non-responders. When the initial control replied late, both the initial and the already enrolled substitute control have been included in the study. This resulted in the inclusion of more controls (n = 1561, men = 1054; women = 507) than cases.

Anthropometric measures were recorded at physical examination and blood samples were collected about three months after the MI [16]. Biochemical measurements were done as previously reported [17]. Family history of CAD was defined as having at least one close relative affected before the age of 65.

### Ethics

The Ethical Committee at Karolinska Institutet approved the SHEEP study design in 1991 (Protocol Number 1991, 91:259). All the study participants gave their informed oral consent to be enrolled in the study, since at the time the study was initiated (1992) no forms for the written consent were available or in current use. The Ethical Committee at Karolinska Institutet has then approved molecular genetic analyses to be performed on the SHEEP material in 2001 (Protocol Number 2001, 01-097).

### Single nucleotide polymorphism (SNP) genotyping

Three SNPs showing the strongest association in the published GWAs studies [6,10] with LDL-serum levels were genotyped and analysed in the present study: two intergenic SNPs, rs599839 and rs646776, and rs12740374 that maps at the 3´UTR of the *CELSR2* gene. Rs599839 was genotyped by Taqman and rs12740374 and rs646776 through the Sequenom iPLEX MassARRAY platforms. Random DNA samples were genotyped twice to check for concordance of genotyping. The call rates were 0.98 (rs599839) and 0.99 (rs12740374 and rs646776).

### Statistical analysis

Continuous traits were expressed as median ± interquartile range (IQTR) and the differences in the distribution of quantitative traits and categorical variables calculated by Kruskal-Wallis and χ2 test, respectively. Kolmogorov-Smirnov test was used to test the normality of the distribution of the lipid serum levels as well as of dependant biomarkers. Pairwise linkage disequilibrium (LD) was estimated by calculation of the r^2^ metric using the software Plink [18]. Concordance to the Hardy-Weinberg equilibrium was tested in cases and controls by the χ2 test with 1DF and threshold p-value of 0.05.

Serum lipid levels were not normally distributed in the SHEEP. To test the effect of the SNPs under investigation on lipid serum levels, a weighted least squares regression, a linear regression analysis that does not assume constant variance for the regression residuals, was used to estimate the regression-coefficient (b) and standard error (SE) under the hypothesis of an additive model, i.e. change in serum levels according to the number of risk alleles (i.e. 00 vs 01 vs 11). To test the association with MI, a logistic regression analysis was performed and odds ratios (OR) with 95% confidence interval (95%CI) were estimated under the assumption of an additive (i.e. 00 vs 01 vs 11), dominant (00 vs 01 + 11) and recessive (00 + 01 vs 11) model of inheritance. The crude ORs (95%CI) were adjusted by age, sex and residential area. Further adjustments including BMI, smoking, hypertension, hypercholesterolemia, hypertriglyceridemia and diabetes mellitus were performed in the adjusted analysis.

The interaction between genotypes and the serum lipid parameters (total-, LDL-cholesterol and ApoB serum levels) was calculated using the biological approach [19]. The biological interaction estimates the difference in the risk, expressed as OR (95%CI), associated with the exposure to only one factor (e.g. ApoB or genotype) and the risk associated with the exposure to both factors as compared to the risk observed in the absence of exposure to both factors. The ratio between the risk observed in the presence of both factors and the risk observed in the reference group can be used to derive the Synergy index (S) [20]. A S > 1 indicates the presence of a synergism while a S < 1 indicates the presence of an antagonism between the two interaction terms [20,21]. In the interaction analysis we have defined the exposure to high serum levels as exposure to serum levels higher or equal to the 75^th^ percentile of total-cholesterol ≥6.6 mmol/L, LDL-cholesterol ≥4.6 mmol/L and ApoB ≥1.7 g/L; the exposure to the genotype as presence of the minor allele versus absence of the minor allele (e.g. AG + GG vs AA). For the purpose of interaction analysis ORs (95%CI) were only adjusted by age, sex and residential area.

Calculations were carried out by SAS (vers 9.1, SAS Institute Inc. Cary, NC).

## Results

Table 1 summarizes the demographic characteristics, serum lipids and biomarkers in the SHEEP study. Men were aged 60 (53-65) and women 61 (54-66). Cardiovascular risk factors were more often observed in cases than in controls. In particular, cases had a higher proportion of hypercholesterolemia than controls (42% vs 30%, p < 0.0001).

**Table 1:** Study population: age, serum lipids, biomarkers and cardiovascular risk factor distribution in cases and controls

	***Cases (N = 1213)***	***Controls (N = 1561)***	***P***
Age (years) M (IQTR)	60 (53-65)	61 (54-66)	0.03
Sex (M/F) (N)	852/361	1054/507	0.06
SBP (mmHg) M (IQTR)	130 (117-145)	140 (125-155)	<0.0001
DPB (mmHg) M (IQTR)	80 (72-86)	82 (77-90)	<0.0001
BMI (kg/m^2^) M (IQTR)	26 (23-28)	25.1 (23-27)	<0.0001
Lipid lowering therapy N(%)	59 (4.8)	55 (3.6)	0.55
*Biochemistry M(IQTR)*			
T-Chol (mmol/L)	6.1 (5.4-6.9)	5.9 (5.2-6.6)	<0.0001
LDL-Chol (mmol/L)	4.2 (3.6-4.8)	3.9 (3.3-4.6)	<0.0001
HDL-Chol (mmol/L)	1 (0.9-1.3)	1.2 (1.0-1.5)	<0.0001
TG (mmol/L)	1.7 (1.2-2.4)	1.3 (0.9-1.8)	<0.0001
ApoA1 (g/L)	1.3 (1.2-1.5)	1.5 (1.3-1.7)	<0.0001
ApoB (g/L)	1.6 (1.4-1.9)	1.4 (1.2-1.7)	<0.0001
*Risk Factors N(%)*			
Family History	400 (32)	358 (23)	<0.0001
Hypertension	561 (46)	817 (53)	0.001
Diabetes	151 (12)	72 (4.7)	<0.0001
Hypercholesterolemia	514 (42)	467 (30)	<0.0001
Hypertriglyceridemia	314 (25)	200 (13)	<0.0001
BMI > 30 kg/m^2^	227 (19)	197 (13)	<0.0001
Current Smokers	580 (48)	455 (29)	<0.0001
Physical Inactivity	523 (43)	536 (34)	0.001

T-Chol = Total-Cholesterol; TG = Triglycerides.

Rs12740374 and rs646776 showed a high degree of pairwise LD (r^2^ = 0.99), while rs599839 was in moderate LD with rs12740374 and rs646776 (both r^2^ = 0.51) therefore only rs646776 and rs599839 were analysed for association.

Genotype and allele frequencies were concordant with those predicted by the Hardy-Weinberg proportions in both cases (rs599839 p = 0.85 and rs646776 p = 0.91) and controls (rs599839 p = 0.30 and rs646776 p = 0.24).

We tested the association of genotypes at rs599839 and rs646776 with lipid serum levels (Table 2). In the presence of the genotype GG at rs599839 and CC at rs646776 lower levels of ApoB, serum total - and LDL-cholesterol were observed. This observation is consistent with published data [1,4-6]where the presence of the G at rs599839 and of the C allele rs646776 were associated with LDL-cholesterol serum levels about 0.2 mmol/L (6-7 mg/dl) lower than the alternate allele and with lower total-cholesterol serum levels [about 0.5 mmol/L (19 mg/dl)]. The effect of each SNP on lipid serum levels is reported in Table 2 and indicates a progressive reduction in total-, LDL-cholesterol and ApoB serum levels associated with the G and the C alleles. 

**Table 2:** Distribution across the genotype strata of total-, LDL-cholesterol and ApoB serum levels and effect of rs599839 and rs646776 on total-, LDL-cholesterol and ApoB serum levels in the SHEEP study

		***Total-Chol*(N = 2646)**	***P***	***LDL-Chol*(N = 2599)**	***P***	***ApoB*(N = 2643)**	***P***
**rs599839**	AA	6.0 (5.3-6.7)		4.1 (3.4-4.7)		1.5 (1.3-1.8)	
	AG	5.8 (5.2-6.6)		3.9 (3.3-4.6)		1.5 (1.3-1.7)	
	GG	5.8 (4.9-6.5)	0.003	3.9 (3.2-4.4)	0.007	1.4 (1.2-1.7)	0.03
**B (SE)**		−0.65 (0.019)	0.001	−0.62 (0.020)	0.002	−0.65(0.019)	0.001
**rs646776**	TT	6.0 (5.3-6.7)		4.1 (3.5-4.7)		1.5 (1.3-1.8)	
	CT	5.9 (5.2-6.7)		3.9 (3.3-4.6)		1.5 (1.2-1.7)	
	CC	5.8 (5.1-6.4)	0.04	3.9 (3.2-4.4)	0.0003	1.4 (1.2-1.7)	0.00001
**B (SE)**		−0.63 (0.019)	0.001	−0.82 (0.020)	<0.0001	−0.83 (0.020)	<0.0001

Total- and LDL-cholesterol serum levels are expressed in mmol/L and ApoB serum levels in g/L. Median ± IQRT are reported at each genotype strata. The effect of each SNP on total-, LDL-cholesterol and ApoB serum levels is reported in the last row of each section and expressed as beta coefficient (b) along with the standard error (SE).

When the analysis was performed in men and women separately, only men consistently showed reduced serum levels of ApoB, total-cholesterol and LDL-cholesterol serum levels (Additional file 1: Table S1).

No significant association of the G at rs599839 as well as of the C allele at rs646776 allele with serum levels of triglycerides, HDL-cholesterol, ApoA1 was observed in men or in women (Additional file 1: Table S2).

No significant differences were observed in genotype and allele frequencies at these two SNPs between MI cases and controls and no association with the risk of MI was observed in this population. Table 3 shows the genotype and allele frequencies of the two SNPs and the analysis of association with the risk of MI under the three different models of inheritance. Allele G frequency at rs599839 was 0.18 in cases and 0.17 in controls, while the allele C frequency at rs646776 was 0.23 in both cases and controls. No association of any of the two SNPs with the risk of MI was observed at the univariate analysis [OR(95%CI)] using three different analytical models, additive [rs599839 1.08(0.93-1.26); rs646776 0.97 (0.85-1.11)], dominant, [rs599839 1.07(0.91-1.27); rs646776 0.95 (0.82-1.11)], and recessive [rs599839 1.36(0.79-2.13); rs646776 1.07 (0.75-1.53)]. Adjustment for the other covariates did not substantial change the risk estimates as shown in Table 3.

**Table 3:** Genotype, allele frequencies and risk of MI associated with rs599839 and rs646776 in the SHEEP

		***MI***		***P***
		**Cases/Controls**		
**rs599839**	AA	779/1035		
	AG	340/431		
	GG	33/33		0.5
***Allele G***		0.18/0.17		0.5
		**Crude**	**Adjusted**	
		**OR (95%CI)**	**OR (95%CI)**	
Additive		1.08 (0.93-1.26)	1.10 (0.93-1.30)	
Dominant		1.07 (0.91-1.27)	1.10 (0.91-1.33)	
Recessive		1.36 (0.79-2.13)	1.23 (0.69-2.17)	
**rs646776**				
	TT	686/875		
	CT	413/561		
	CC	58/70		0.7
***Allele C***		0.23/0.23		0.8
		**Crude**	**Adjusted**	
		**OR (95%CI)**	**OR (95%CI)**	
Additive		0.97 (0.85-1.11)	0.97 (0.84-1.13)	
Dominant		0.95 (0.82-1.11)	0.94 (0.78-1.12)	
Recessive		1.07 (0.75-1.53)	1.15 (0.76-1.73)	

**Figure 1:**
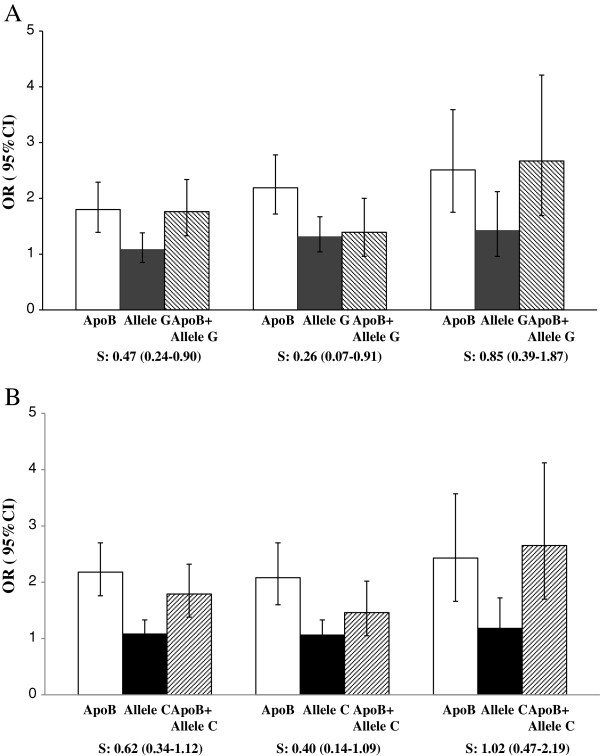
**Top panel** (**A**)**.** Biological interaction (left to right striped bars) between the exposure to high (≥75^th^ percentile) ApoB serum levels (white bars) and the presence of the rare allele at rs599839 (gray bars) in all SHEEP participants (left), in men (middle) and in women (right). Bottom panel (**B**). Biological interaction (right to left striped bars) between the exposure to high (≥75^th^ percentile) ApoB serum levels (white bars) and the presence of the rare allele and at rs646776 (black bars), in all SHEEP participants (left), in men (middle) and in women (right). The reference group is represented by the individuals not exposed to ApoB serum levels nor to the G or C alleles. Error bars indicate the 95%CI; S: Sinergy Index.

We have then tested the hypothesis that, in the SHEEP, the interaction between the genetic variants at 1p13 and serum lipid levels was an important player in explaining the lack of association between the 1p13 genetic variants and the MI risk. Given the causal association between serum lipid levels and MI, we analysed the interaction between serum lipid levels and genotypes using the biological approach. As reported in Table 4 and Figure 1 (top panel), presence of the allele G at rs599839 antagonizes the risk associated with the exposure to high (≥75^th^ percentile) serum levels of ApoB. The calculated S of 0.47 (0.24-0.90) indicates antagonism between the two interaction terms. When the analysis was performed in men and women the protective effect of the rare allele was observed only in men with a S of 0.26 (0.07-0.91) (Figure 1, top panel, middle bar graph). The actual ORs (95%CI) for the gender specific interaction analysis are reported in the Additional file 1: Table S3. A trend toward a reduction in MI risk in men carrying the G allele and exposed to increased total- and LDL cholesterol levels was also observed with a S lower than 1, however the results fell short of statistical significance (Table 4).

**Table 4:** Effect of interaction between serum lipid levels and genetic variants at 1p13 on the risk of MI expressed as Odds Ratio (OR) and 95%CI in the SHEEP

		**N**	**OR (95%CI)**		**N**	**OR (95%CI)**		**N**	**OR (95%CI)**
**Rs599839**	ApoB ≥75^th^ perc	617	2.27	Tot-chol ≥75^th^ perc	611	1.72	LDL-chol ≥75^th^ perc	1304	1.63
			(1.86-2.77)			(1.40-2.10)			(1.32-2.01)
	AG + GG vs AA	601	1.34	AG + GG vs AA	562	1.13	AG + GG vs AA	188	1.13
			(1.10-1.64)			(0.93-1.37)			(0.94-1.37)
	Both	234	1.76	Both	474	1.80	Both	474	1.68
			(1.33-2.34)			(1.36-2.37)			(1.24-2.27)
S (95%CI)			0.47			0.94			0.89
			(0.24-0.90)			(0.46-1.92)			(0.38-2.06)
**Rs646776**	ApoB ≥75^th^ perc	536	2.18	Tot-chol ≥75th perc	582	1.79	LDL-chol ≥75th perc	416	1.54
			(1.76-2.70)			(1.45-2.20)			(1.23-1.93)
	CC + CT vs TT	787	1.09	CC + CT vs TT	751	0.98	CC + CT vs TT	837	0.94
			(0.90-1.33)			(0.81-1.19)			(0.79-1.13)
	Both	312	1.79	Both	350	1.76	Both	243	1.68
			(1.38-2.32)			(1.37-2.25)			(1.27-2.23)
S (95%CI)			0.62			0.97			1.39
			(0.34-1.12)			(0.50-1.89)			(0.53-3.63)

The table provides the OR (95%CI) relative to the risk of MI associated with the exposure to high serum levels of ApoB, total-cholesterol (tot-chol), LDL-cholesterol (LDL-chol) in the absence of the allele G at rs599839 and of the allele C at rs646776 in the first row of the two sections; the risk associated with the exposure to the allele G or C in the absence of the exposure to high lipid serum levels in the second row of each section and the risk associated with the exposure to both serum levels and genotype in the third row of each section. The last row of each section reports the S index along with the 95%CI.

The analysis of the interaction between high ApoB serum levels and the C allele at rs646776 also suggested the presence of an antagonistic effect with and S index of 0.62 (0.34-1.12) (Table 4 and Figure 1, bottom panel) that was also confirmed in men with a S 0.40 (0.14-1.09), but with a larger 95%CI (Additional file 1: Table S2 and Figure 1 bottom panel).

## Discussion

The intergenic SNPs rs599839 and rs646776 have been identified through GWAs as novel genetic markers for two complex and related traits, serum lipid levels and CAD. In the present study, performed in the SHEEP, a large Swedish population, we confirmed the association of these two genetic variants with serum lipid levels; however we have not observed a direct association between these two genetic variants and the risk of non-fatal MI. We have therefore tested the hypothesis that the interaction between these two SNPs and serum lipid levels contributed to the risk of non-fatal MI in the SHEEP.

The analysis of the association of genetic variants with complex phenotypes may largely vary among different populations. Genes do not have large effect on complex traits and differences in the definition of the trait under investigation as well as the genetic structure of the loci may create large differences in the association results. Although the lack of association in the SHEEP population might partly reflect a reduced power in the association analysis as compared to the analysis of genetic association in large international consortia, several other factors should be taken into account. In the populations formerly investigated different criteria have been used to identify to cases, the phenotype under investigation was either CAD [6] or early myocardial infarction in patients with at least one first degree relative with premature CAD [1] and the matching criteria for the controls were sometimes incompletely described [6,9]. In the current study, only MI patients who survived at least 28 days after the MI event have been included and the referent population has been matched according to age, sex and residential area. Therefore lack of direct association of 1p13 variants with MI in the SHEEP might be related to the differences in the definition of cases as well as to the controls selection. In addition, differences in the genetic structure of the populations under investigation may hamper the replication of genetic associations. With regard to the chromosome 1p13 locus we have observed that in the SHEEP the pairwise LD value between rs599839 and rs646776 is different from the one currently reported in the Hapmap Consortium ( http://www.hapmap.org) for the European population (r^2^ = 0.51 observed vs 0.87 reported). These data speak in favor of a different genetic structure of this locus in the Swedish population and are consistent with the hypothesis raised by evolutionary geneticists stating that European populations have a composite genetic structure due to recent gene selection events (10 000-20 000 years ago) that might have changed pairwise LD values [22]. In addition, the G allele frequency at rs599839 in the SHEEP (17%) is lower than previously reported in former studies (23%) [2,4,5] and in the European panel of the HapMap (33%). Such findings underscore the importance of the knowledge of the locus structure when analysing the effect of genetic variants on a phenotype even within populations of the same ethnicity [17,23-25] and may well explain discrepancies in the genetic effect of even truly genetic susceptibility variants [12,26].

In the SHEEP, the risk of MI was increased in the presence of high ApoB serum levels and presence of the rare allele at rs599839, and to a lesser extent of the rare allele at rs647767, was found to antagonize the increased risk due to the exposure to high ApoB serum levels, as shown by the results of the interaction analysis. Although we cannot provide proof of a biological mechanism, this interpretation is in line with the results of the original GWAs studies, where the protective effect of 1p13 was observed in populations where the proportion of cases with dyslipidemia ranged from 76 to 80% [4,6] and was therefore higher than the proportion reported in the SHEEP that is about 40%.

The analysis of interaction represents a powerful tool to integrate genetic association data into the complexity of multifactorial traits [19]. In the present study we have utilized the biological method to analyze the effect of the interaction between genetic variants at 1p13 and serum lipid levels because they participate in the same causal mechanism that leads to MI. The elucidation of the mechanisms underlying interactions between genetic variants and environmental factors or, as in the present study exposure that may be modulated by pharmacological interventions, might have important implication in the assessment of the individual cardiovascular risk as well as in the clinical practice. Exposure to a specific agent may in fact have more or less detrimental effect in different genotype groups if an interaction between the genotype and the exposure exists [27]. In this perspective gene environment interaction analyses hold the promise to contribute to a better understanding of the effect of genetic variants on the risk of cardiovascular diseases.

The association with reduced serum lipid levels was evident only in men. A gender specific association of genetic variants with MI and intermediate phenotypes has already been reported in the SHEEP [17,28] and may reflect the selective effect of risk factors in men and women [29].

Several limitations of the present study should be acknowledged. The choice of the SNPs to be investigated in the present studies relies on published data and does not include the other two tagSNPs, rs4970834 and rs611917, at chromosome1p13. The interaction analyses may be hard to interpreter and require large study population to achieve a sufficient power, therefore the replication of our findings in a larger and independent population is warranted.

## Conclusions

In conclusion, our results demonstrate that genetic variants at chromosome 1p13 reduce the MI risk in this Swedish population mainly through the interaction with ApoB serum levels, thus supporting the evidence for a causal role of this locus in the occurrence of MI.

## Competing interests

The authors declare no conflict of interest.

## Authors’ contributions

BG has made substantial contributions to conception and design, analysis and interpretation of data; has drafted and revised the manuscript; KL has made substantial contributions to analysis and interpretation of data; has been involved in revising critically the manuscript for important intellectual content; MV has made substantial contributions to analysis and interpretation of data; SY has made substantial contributions to acquisition of data, analysis and interpretation of data; has been involved in revising the manuscript critically for important intellectual content; UdF has made substantial contributions to conception and design of the study, analysis and interpretation of data; has been involved revising the manuscript critically for important intellectual content. All Authors have given final approval of the manuscript version to be published.

## Pre-publication history

The pre-publication history for this paper can be accessed here:

http://www.biomedcentral.com/1471-2261/12/90/prepub

## Supplementary Material

Additional file 1 **Table S1.** Total-, LDL-cholesterol, ApoB, serum levels according to genotype at rs599839 and rs646776 in men and women. **Table S2.** Serum levels of HDL-cholesterol, ApoA1 and triglycerides (TG) according to genotype at rs599839 and rs646776 in the SHEEP population. **Table S3.** Interaction analysis: Risk of MI expressed as OR and 95%CI associated with the exposure to ApoB serum levels, the rare allele at rs599839 and rs646776 and the interaction term in men and women. Click here for file

## Author details

^1^Division of Cardiovascular Epidemiology, Institute of Environmental Medicine (IMM), Karolinska Institutet, Stockholm, Sweden. ^2^Division of Cardiovascular Medicine, Department of Clinical Sciences, Danderyd Hospital, Karolinska Institutet, Stockholm, Sweden. ^3^Department of Cardiology, Karolinska University Hospital, Stockholm, Sweden. ^4^Centre of Clinical Pharmacology, William Harvey Research Institute, Barts and the London School of Medicine and Dentistry, Queen Mary University of London, London, UK.

## References

[B1] Karvanen J Silander K Kee F Tiret L Salomaa V Kuulasmaa K Wiklund PG Virtamo J Saarela O Perret C The impact of newly identified loci on coronary heart disease, stroke and total mortality in the MORGAM prospective cohorts Genet Epidemiol 2009 33 3 237 246 10.1002/gepi.20374 18979498 PMC2696097

[B2] Kathiresan S Melander O Anevski D Guiducci C Burtt NP Roos C Hirschhorn JN Berglund G Hedblad B Groop L Polymorphisms associated with cholesterol and risk of cardiovascular events N Engl J Med 2008 358 12 1240 1249 10.1056/NEJMoa0706728 18354102

[B3] Kathiresan S Melander O Guiducci C Surti A Burtt NP Rieder MJ Cooper GM Roos C Voight BF Havulinna AS Six new loci associated with blood low-density lipoprotein cholesterol, high-density lipoprotein cholesterol or triglycerides in humans Nat Genet 2008 40 2 189 197 10.1038/ng.75 18193044 PMC2682493

[B4] Samani NJ Braund PS Erdmann J Gotz A Tomaszewski M Linsel-Nitschke P Hajat C Mangino M Hengstenberg C Stark K The novel genetic variant predisposing to coronary artery disease in the region of the PSRC1 and CELSR2 genes on chromosome 1 associates with serum cholesterol J Mol Med (Berl) 2008 86 11 1233 1241 10.1007/s00109-008-0387-2 18649068

[B5] Sandhu MS Waterworth DM Debenham SL Wheeler E Papadakis K Zhao JH Song K Yuan X Johnson T Ashford S LDL-cholesterol concentrations: a genome-wide association study Lancet 2008 371 9611 483 491 10.1016/S0140-6736(08)60208-1 18262040 PMC2292820

[B6] Willer CJ Sanna S Jackson AU Scuteri A Bonnycastle LL Clarke R Heath SC Timpson NJ Najjar SS Stringham HM Newly identified loci that influence lipid concentrations and risk of coronary artery disease Nat Genet 2008 40 2 161 169 10.1038/ng.76 18193043 PMC5206900

[B7] Linsel-Nitschke P Heeren J Aherrahrou Z Bruse P Gieger C Illig T Prokisch H Heim K Doering A Peters A Genetic variation at chromosome 1p13.3 affects sortilin mRNA expression, cellular LDL-uptake and serum LDL levels which translates to the risk of coronary artery disease Atherosclerosis 2010 208 1 183 189 10.1016/j.atherosclerosis.2009.06.034 19660754

[B8] Eagle KA Ginsburg GS Musunuru K Aird WC Balaban RS Bennett SK Blumenthal RS Coughlin SR Davidson KW Frohlich ED Identifying patients at high risk of a cardiovascular event in the near future: current status and future directions: report of a national heart, lung, and blood institute working group Circulation 2010 121 12 1447 1454 10.1161/CIRCULATIONAHA.109.904029 20351302 PMC2905873

[B9] Samani NJ Erdmann J Hall AS Hengstenberg C Mangino M Mayer B Dixon RJ Meitinger T Braund P Wichmann HE Genomewide association analysis of coronary artery disease N Engl J Med 2007 357 5 443 453 10.1056/NEJMoa072366 17634449 PMC2719290

[B10] Kathiresan S Voight BF Purcell S Musunuru K Ardissino D Mannucci PM Anand S Engert JC Samani NJ Schunkert H Genome-wide association of early-onset myocardial infarction with single nucleotide polymorphisms and copy number variants Nat Genet 2009 41 3 334 341 10.1038/ng.327 19198609 PMC2681011

[B11] Ioannidis JP Prediction of cardiovascular disease outcomes and established cardiovascular risk factors by genome-wide association markers Circ Cardiovasc Genet 2009 2 1 7 15 10.1161/CIRCGENETICS.108.833392 20031562

[B12] Greene CS Penrod NM Williams SM Moore JH Failure to replicate a genetic association may provide important clues about genetic architecture PLoS One 2009 4 6 e5639 10.1371/journal.pone.0005639 19503614 PMC2685469

[B13] Zondervan KT Cardon LR Designing candidate gene and genome-wide case-control association studies Nat Protoc 2007 2 10 2492 2501 10.1038/nprot.2007.366 17947991 PMC4180089

[B14] Manolio TA Collins FS Cox NJ Goldstein DB Hindorff LA Hunter DJ McCarthy MI Ramos EM Cardon LR Chakravarti A Finding the missing heritability of complex diseases Nature 2009 461 7265 747 753 10.1038/nature08494 19812666 PMC2831613

[B15] Sanna S Li B Mulas A Sidore C Kang HM Jackson AU Piras MG Usala G Maninchedda G Sassu A Fine mapping of five loci associated with low-density lipoprotein cholesterol detects variants that double the explained heritability PLoS Genet 2011 7 7 e1002198 10.1371/journal.pgen.1002198 21829380 PMC3145627

[B16] Reuterwall C Hallqvist J Ahlbom A De Faire U Diderichsen F Hogstedt C Pershagen G Theorell T Wiman B Wolk A Higher relative, but lower absolute risks of myocardial infarction in women than in men: analysis of some major risk factors in the SHEEP study. The SHEEP Study Group J Intern Med 1999 246 161 174 10.1046/j.1365-2796.1999.00554.x 10447785

[B17] Gigante B Vikstrom M Meuzelaar LS Chernogubova E Silveira A Hooft FV Hamsten A de Faire U Variants in the coagulation factor 2 receptor (F2R) gene influence the risk of myocardial infarction in men through an interaction with interleukin 6 serum levels Thromb Haemost 2009 101 5 943 953 19404549

[B18] Purcell S Neale B Todd-Brown K Thomas L Ferreira MA Bender D Maller J Sklar P de Bakker PI Daly MJ PLINK: a tool set for whole-genome association and population-based linkage analyses Am J Hum Genet 2007 81 3 559 575 10.1086/519795 17701901 PMC1950838

[B19] Gigante B Bennet AM Leander K Vikstrom M de Faire U The interaction between coagulation factor 2 receptor and interleukin 6 haplotypes increases the risk of myocardial infarction in men PLoS One 2010 5 6 e11300 10.1371/journal.pone.0011300 20585578 PMC2891999

[B20] Rothman KJ The estimation of synergy or antagonism Am J Epidemiol 1976 103 5 506 511 1274952 10.1093/oxfordjournals.aje.a112252

[B21] Lundberg M Fredlund P Hallqvist J Diderichsen F A SAS program calculating three measures of interaction with confidence intervals Epidemiology 1996 7 6 655 656 8899400

[B22] Schmegner C Hoegel J Vogel W Assum G A comparison of the variability spectra of two genomic loci in a European group of individuals reveals fundamental differences pointing to selection or a population bottleneck Ann Hum Genet 2007 71 Pt 3 370 378 17222291 10.1111/j.1469-1809.2006.00342.x

[B23] Liu YJ Papasian CJ Liu JF Hamilton J Deng HW Is replication the gold standard for validating genome-wide association findings? PLoS One 2008 3 12 e4037 10.1371/journal.pone.0004037 19112512 PMC2605260

[B24] Jakkula E Rehnstrom K Varilo T Pietilainen OP Paunio T Pedersen NL de Faire U Jarvelin MR Saharinen J Freimer N The genome-wide patterns of variation expose significant substructure in a founder population Am J Hum Genet 2008 83 6 787 794 10.1016/j.ajhg.2008.11.005 19061986 PMC2668058

[B25] Salmela E Lappalainen T Liu J Sistonen P Andersen PM Schreiber S Savontaus ML Czene K Lahermo P Hall P Swedish population substructure revealed by genome-wide single nucleotide polymorphism data PLoS One 2011 6 2 e16747 10.1371/journal.pone.0016747 21347369 PMC3036708

[B26] Altshuler D Daly MJ Lander ES Genetic mapping in human disease Science 2008 322 5903 881 888 10.1126/science.1156409 18988837 PMC2694957

[B27] Dempfle A Scherag A Hein R Beckmann L Chang-Claude J Schafer H Gene-environment interactions for complex traits: definitions, methodological requirements and challenges Eur J Hum Genet 2008 16 10 1164 1172 10.1038/ejhg.2008.106 18523454

[B28] Zotova E Lyrenas L de Faire U Morgenstern R Gigante B Bennet AM The myeloperoxidase gene and its influence on myocardial infarction in a Swedish population: protective role of the -129A allele in women Coron Artery Dis 2009 20 5 322 326 10.1097/MCA.0b013e32832da06d 19543083

[B29] Burke AP Farb A Malcom GT Liang Y Smialek J Virmani R Effect of risk factors on the mechanism of acute thrombosis and sudden coronary death in women Circulation 1998 97 21 2110 2116 10.1161/01.CIR.97.21.2110 9626170

