# Supplementary material for: Chromosome 1p13 genetic variants antagonize the risk of myocardial infarction associated with high ApoB serum levels
Source: BMC Cardiovasc Disord. 2012 Oct 16;12:90. doi: 10.1186/1471-2261-12-90 (PMC3480949; doi:10.1186/1471-2261-12-90)
Supplement: Additional file 1 — Table S1. Total-, LDL-cholesterol, ApoB, serum levels according to genotype at rs599839 and rs646776 in men and women. Table S2. Serum levels of HDL-cholesterol, ApoA1 and triglycerides (TG) according to genotype at rs599839 and rs646776 in the SHEEP population. Table S3. Interaction analysis: Risk of MI expressed as OR and 95%CI associated with the exposure to ApoB serum levels, the rare allele at rs599839 and rs646776 and the interaction term in men and women. [file 1471-2261-12-90-S1.doc]

**Online Supplemental material to the article entitled “Chromosome 1p13 genetic variants antagonize the risk of myocardial infarction associated with high ApoB serum levels.”**

**Supplemental Table I.** Total-, LDL-cholesterol, ApoB, serum levels according to genotype at rs599839 and rs646776 in men and women.

|  |  | **Men** |  | **Women** |  |
| --- | --- | --- | --- | --- | --- |
| **Total-Chol** |  | (N=1826) | **P** | (N=820) | **P** |
| rs599839 | AA | 5.9 (5.3-6.6) |  | 6.2 (5.5-7.0) |  |
|  | AG | 5.7 (4.7-6.5) |  | 6.2 (5.5-6.9) |  |
|  | GG | 5.6 (4.7-6.5) | 0.001 | 6.1 (5.1-6.7) | 0.62 |
| rs646776 | TT | 5.9 (5.3-6.7) |  | 6.2 (5.5-7.1) |  |
|  | CT | 5.8 (5.1-6.5) |  | 6.2 (5.5-7.0) |  |
|  | CC | 5.8 (4.9-6.4) | 0.003 | 6.1 (5.2-6.4) | 0.17 |
| **LDL-Chol** |  | (N=1788) |  | (N=811) |  |
| rs599839 | AA | 4.1 (3.4-4.6) |  | 4.1 (3.4-4.9) |  |
|  | AG | 3.9 (3.3-4.6) |  | 4.1 (3.5-4.8) |  |
|  | GG | 3.7 (3.1-4.6) | 0.004 | 3.9 (3.2-4.5) | 0.57 |
| rs646776 | TT | 4.1 (3.5-4.7) |  | 4.1 (3.4-4.9) |  |
|  | CT | 3.8 (3.3-4.6) |  | 4.1 (3.5-4.8) |  |
|  | CC | 3.9 (3.1-4.4) | 0.0002 | 3.9 (3.3-4.4) | 0.18 |
| **ApoB** |  | (N=1824) |  | (N=819) |  |
| rs599839 | AA | 1.5 (1.3-1.8) |  | 1.5 (1.3-1.8) |  |
|  | AG | 1.5 (1.2-1.7) |  | 1.6 (1.3-1.9) |  |
|  | GG | 1.3 (1.2-1.7) | 0.0002 | 1.5 (1.2-1.8) | 0.50 |
| rs646776 | TT | 1.5 (1.3-1.8) |  | 1.6 (1.3-1.8) |  |
|  | CT | 1.5 (1.2-1.7) |  | 1.5 (1.3-1.9) |  |
|  | CC | 1.4 (1.2-1.6) | 0.0001 | 1.5 (1.2-1.7) | 0.31 |
|  |  |  |  |  |  |

**Supplemental Table II**. Serum levels of HDL-cholesterol, ApoA1 and triglycerides (TG) according to genotype at rs599839 and rs646776 in the SHEEP population.

|  |  | All |  | Men |  | Women |  |
| --- | --- | --- | --- | --- | --- | --- | --- |
| **HDL-Chol** |  | (N=2625) |  | (N=1812) |  | (N=813) |  |
| rs599839 | AA | 1.1 (0.9-1.4) |  | 1.1 (0.9-1.3) |  | 1.3 (1.1-1.6) |  |
|  | AG | 1.1(0.9-1.4) |  | 1.1 (0.9-1.3) |  | 1.3 (1.1-1.4) |  |
|  | GG | 1.2 (1-1.4) | 0.58 | 1.1 (0.9-1.3) | 0.92 | 1.4 (1.1-1.6) | 0.89 |
| rs646776 | TT | 1.1 (0.9-1.4) |  | 1.1 (0.9-1.3) |  | 1.3 (1.1-1.6) |  |
|  | CT | 1.1 (0.9-1.4) |  | 1.0 (0.9-1.3) |  | 1.3 (1.1-1.6) |  |
|  | CC | 1.2 (1.0-1.4) | 0.14 | 1.2 (1.0-1.3) | 0.03 | 1.3 (1.1-1.6) |  |
| **ApoA1** |  | (N=2643) |  | (N=1824) |  | (N=819) |  |
| rs599839 | AA | 1.4 (1.3-1.6) |  | 1.4 (1.2-1.6) |  | 1.5 (1.4-1.7) |  |
|  | AG | 1.4 (1.3-1.6) |  | 1.4 (1.2-1.6) |  | 1.6 (1.3-1.8) |  |
|  | GG | 1.5 (1.3-1.6) | 0.79 | 1.4 (1.2-1.6) | 0.87 | 1.6 (1.4-1.7) | 0.95 |
| rs646776 | TT | 1.4 (1.3-1.6) |  | 1.4 (1.2-1.6) |  | 1.6 (1.4-1.7) |  |
|  | CT | 1.4 (1.3-1.6) |  | 1.4 (1.3-1.5) |  | 1.6 (1.4-1.7) |  |
|  | CC | 1.5 (1.3-1.7) | 0.19 | 1.4 (1.2-1.6) | 0.07 | 1.6 (1.3-1.7) |  |
| **TG** |  | (N=2648) |  | (N=1827) |  | (N=821) |  |
| rs599839 | AA | 1.4 (1.0-2.1) |  | 1.5 (1.1-2.1) |  | 1.4 (1.0-2.1) |  |
|  | AG | 1.4 (1.1-2.0) |  | 1.4 (1.1-2.1) |  | 1.4 (1.0-2.0) |  |
|  | GG | 1.5 (1.0-1.8) | 0.84 | 1.5 (1.0-1.8) | 0.50 | 1.5 (1.1-1.9) | 0.76 |
| rs646776 | TT | 1.4 (1.0-2.1) |  | 1.5 (1.1-2.1) |  | 1.4 (1.0-2.0) |  |
|  | CT | 1.4 (1.0-2.1) |  | 1.5 (1.1-2.1) |  | 1.4 (1.0-1.9) |  |
|  | CC | 1.5 (1.0-1.9) | 0.78 | 1.4 (1.0-1.3) | 0.22 | 1.7 (1.1-2.1) |  |

**Supplemental Table III.** Interaction analysis: Risk of MI expressed as OR and 95%CI associated with the exposure to ApoB serum levels, the rare allele at rs599839 and rs646776 and the interaction term in men and women.

|  | **N** | **All OR (95%CI)** | **N** | **Men OR (95%CI)** | **N** | **Women OR (95%CI)** |
| --- | --- | --- | --- | --- | --- | --- |
| **Rs599839** |  |  |  |  |  |  |
| ApoB ≥75^th^ perc | 617 | 2.27(1.86-2.77) | 406 | 2.19 (1.72-2.78) | 211 | 2.51 (1.75-3.59) |
| AG+GG vs AA | 601 | 1.34 (1.10-1.64) | 439 | 1.32 (1.04-1.67) | 162 | 1.43 (0.96-2.12) |
| Both | 234 | 1.76 (1.33-2.34) | 137 | 1.39 (0.96-2.00) | 97 | 2.67 (1.69-4.21) |
| **Rs646776** |  |  |  |  |  |  |
| ApoB ≥75^th^ perc | 536 | 2.18 (1.76-2.70) | 351 | 2.08 (1.60-2.7) | 185 | 2.43 (1.66-3.57) |
| CC+CT vs TT | 787 | 1.09 (0.90-1.33) | 568 | 1.07 (0.85-1.33) | 219 | 1.19 (0.82-1.72) |
| Both | 312 | 1.79 (1.38-2.32) | 189 | 1.46 (1.05-2.02) | 124 | 2.65 (1.70-4.12) |

ApoB ≥75^th^ perc: risk associated with the exposure to ApoB serum levels ≥75^th^ percentile in individuals not carrying the G or the C allele

GG+AG vs GG and CC+CT vs TT: risk associated with the exposure to either the G or the C allele in individuals not exposed to high ApoB serum levels

Both: risk estimate in individuals exposed to both high ApoB serum levels and the G or the C allele.

The reference group is represented by the individuals not exposed to ApoB serum levels nor to the G or C alleles.
